# Induction of retinol-binding protein 4 and placenta-specific 8 expression in human prostate cancer cells remaining in bone following osteolytic tumor growth inhibition by osteoprotegerin

**DOI:** 10.3892/ijo.2013.1954

**Published:** 2013-05-24

**Authors:** HISANORI UEHARA, TETSUYUKI TAKAHASHI, KEISUKE IZUMI

**Affiliations:** Department of Molecular and Environmental Pathology, Institute of Health Biosciences, The University of Tokushima Graduate School, Tokushima-shi, Tokushima 770-8503, Japan

**Keywords:** osteoprotegerin, retinol binding protein 4, placenta-specific 8, bone metastasis, prostate cancer

## Abstract

New drugs that inhibit the osteoprotegerin (OPG)/receptor activator of NF-κB ligand (RANKL)/RANK pathway have demonstrated efficacy for the treatment of bone metastasis. Toxicities induced by these drugs, however, including osteonecrosis of the jaw and hypocalcemia, may adversely affect therapy. The aim of this study was to identify additional therapeutic targets that can be combined with OPG/RANKL/RANK pathway inhibition in the treatment of prostate cancer bone metastasis. We established a stable transfectant that produces high levels of OPG mRNA and protein from PC-3 human prostate cancer cells (PC3-OPG). The culture medium of PC3-OPG cells significantly inhibited the differentiation of mouse monocytes into mature osteoclasts. Furthermore, when PC3-OPG cells were injected into the bones of nude mice, bone destruction and tumor-induced osteoclast formation were reduced. Injection into bone of the mixtures containing equal amounts of green fluorescent protein (GFP)-expressing PC-3 cells (PC3-GFP) and PC3-OPG cells also reduced bone destruction, compared to the control mixture. PC3-GFP cells were subsequently isolated from bone tumors and used for microarray analysis to assess changes in gene expression following osteolytic tumor growth inhibition by OPG. We selected the top 10 upregulated genes based on results from microarrays and confirmed mRNA expression of each gene by RT-PCR. The expression patterns of retinol-binding protein 4 (RBP4) and placenta-specific 8 (PLAC8) were consistent with microarray results. Expression of these genes was also increased in the bone tumors of PC3-GFP/PC3-OPG-injected mice. Knockdown of both RBP4 and PLAC8 by siRNA inhibited the growth of PC-3 cells *in vitro*. Thus, RBP4 and PLAC8 may become new therapeutic targets for prostate cancer bone metastasis, in combination with OPG/RANKL/RANK pathway inhibition.

## Introduction

Prostate cancer continues to be the most common cancer and the second leading cause of cancer deaths in the United States. It is estimated that 240,890 men were diagnosed with the disease and 33,730 died of it in 2011 (1). The skeleton is the most common site of distant metastasis in prostate cancer and bone metastasis was detected at autopsy in up to 90% of patients that died of the disease (2). Although metastatic lesions of prostate cancer in bone are predominantly osteoblastic, they can also be mixed or entirely osteolytic (3–6).

Skeletal-related events (SREs), such as pathologic fractures, spinal cord compression, surgery to bone and radiation to bone that occur frequently in patients with bone metastases, contribute substantially to morbidity and mortality and can reduce health-related quality of life. Management of bone metastases is therefore an important issue in the treatment of prostate cancer (7,8).

Disruption of homeostasis in the bone microenvironment plays a pivotal role in metastasis and tumor growth in bone. One key event is alteration of the bone remodeling system, which is closely related to bone metastasis (9). Three main factors associated with bone remodeling are receptor activator of NF-κB ligand (RANKL), RANK and osteoprotegerin (OPG). RANKL on the surface of osteoblasts binds to RANK on osteoclast progenitors and stimulates differentiation, activation and survival of these cells by recruiting tumor necrosis factor (TNF)-associated family of proteins, which act as cytosolic adaptor proteins (10). OPG, a 55-kDa secretory glycoprotein belonging to the TNF receptor superfamily, acts as a decoy receptor for RANKL and inhibits osteoclastogenesis and ovariectomy-induced bone loss (11). Mice lacking OPG were shown to exhibit a decrease in total bone density and arterial calcification (12).

RANKL inhibition has been studied in both *in vitro* and *in vivo* models of prostate cancer. In one study, treatment of mice with recombinant mouse OPG protein inhibited prostate tumor-induced osteoclastogenesis and tumor growth in bone but had no effect on subcutaneous tumor growth, suggesting the absence of a direct antitumor effect (13). Similarly, when OPG-overexpressing C4-2 CaP cells were injected intraosseously into immunodeficient mice, a reduction in tumor-burden was observed, although no effect on tumor growth was seen when these cells were grown subcutaneously (14). Treatment with RANK.Fc inhibited osteoblastic growth of LuCaP35 cells growing in the bone of SCID mice (15). Collectively, these reports suggest that the OPG/RANKL/RANK pathway is a good molecular target for prevention of prostate cancer bone metastasis.

In addition to its role in regulating tumor-induced bone disease, however, the RANKL system may be associated with other distinct biological effects. For example, OPG may protect tumor cells from apoptosis induced by TRAIL (16,17). In addition, there are data showing that OPG positively regulates microvessel formation, whereas RANKL acts as angiogenic inhibitor (18). Thus, the RANKL system is complex. Furthermore, drugs used for the treatment of bone metastasis, which inhibit the OPG/RANKL/RANK pathway, have been reported to cause other toxicities including osteo-necrosis of the jaw and hypocalcemia (19,20). Since these results of treatment may have adverse effects on therapy, it is necessary to identify additional therapeutic targets that can be combined with OPG/RANKL/RANK pathway inhibition in the treatment of bone metastasis.

In the present study, we established a stable transfectant that produces and secretes a high level of OPG protein from PC-3 human prostate cancer cells (PC3-OPG) and investigated its *in vitro* and *in vivo* characteristics. In addition, mixtures containing equal amounts of green fluorescent protein (GFP)-expressing PC-3 cells (PC3-GFP) and PC3-OPG or PC3-mock were injected into the bones of nude mice. PC3-GFP cells were subsequently isolated from bone tumors and used for micro-array analysis to assess changes in gene expression following osteolytic tumor growth inhibition by OPG. The effects of knockdown of two upregulated genes were also examined in PC-3 cells. The overall goal of this study was to identify additional therapeutic targets that can be used in combination with OPG/RANKL/RANK pathway inhibition in the treatment of prostate cancer bone metastasis.

## Materials and methods

### Cell culture

The human prostate adenocarcinoma cell line PC-3 was maintained in MEM supplemented with 10% fetal bovine serum, 100 U/ml of penicillin G and 0.1 mg/ml streptomycin sulfate.

### Animals

Four-week-old male athymic nude mice were purchased from Charles River Japan, Inc. (Yokohama, Japan). The mice were housed and maintained under specific pathogen-free conditions. Experiments were performed according to the Guideline for the Care and Use of Laboratory Animals of the University of Tokushima School of Medicine and all experimental protocols were approved by the Animal Committee.

### Construction of expression vectors and transfection

The mammary expression vectors, pIRESneo3 and pAcGFP-C1 were purchased from Clonetech Inc. (Mountain View, CA, USA). Human OPG cDNA was obtained by reverse-transcription polymerase chain reaction (RT-PCR) of total RNA from PC-3 cells. Reverse-transcription was conducted at 42°C for 60 min, after which the temperature was increased to 72°C for 15 min, using SuperScript II reverse transcriptase and random hexamers (Invitrogen, Carlsbad, CA, USA). The obtained total cDNA was then amplified by polymerase chain reaction (PCR) following a thermal cycling program of 94°C for 10 min for initial denaturation, 40 cycles of 94°C for 30 sec, 55°C for 1 min and 72°C for 1 min for amplification and a final extension at 72°C for 10 min. Specific primers for hOPG were designed as follows: Eco-hOPG-F (sense) 5′-GAATTCATGAACAA GTTGCTGTGC-3′, Not-hOPG-R (antisense) 5′-GCGGCCGC CCATTTCCAGTTATAAGCAGC-3′. The OPG cDNA fragment was subcloned into the pIRESneo3 vector at the *Eco*RI and *Not*I restriction sites and the resulting vector was designated as pIRES-OPG. Sequences were further confirmed using an ABI PRISM^®^ 3100-Avant Genetic Analyzer (Applied Biosystems, Foster City, CA, USA).

A dose of 13.6 *μ*g of control pIRESneo3, pIRES-OPG or pAcGFP-C1 vectors was introduced into PC-3 cells (4×10^5^ cells/100-mm dish) using TransFast™ transfection reagent (Promega, Madison, WI, USA) according to the manufacturer’s protocol. Transfectants were cultured in complete medium for 48 h and then selected for 72 h with 1 mg/ml of G418 sulfate (Promega). After selection, G418-resistant cells were routinely maintained in a medium containing 0.1 mg/ml G418. These stable transfectants were designated as PC3-mock, PC3-OPG and PC3-GFP, respectively.

### Semi-quantitative RT-PCR

OPG mRNA levels were determined by semi-quantitative RT-PCR. Total RNA was isolated using an RNeasy^®^ Mini kit (Qiagen GmbH, Hilden, Germany) from 5×10^5^ PC3-mock or PC3-OPG cells. After quantification of the RNA concentration, 1 *μ*g of total RNA was subjected to RT-PCR. Reaction conditions were the same as described in ‘Construction of expression vectors and transfection’, except for the number of amplification cycles (30 cycles). In addition to OPG, β-actin was also amplified as an internal standard. The sequences of specific primers for β-actin were as follows: β-actin-F (sense) 5′-TAC AAT GAG CTG CGT GTGG-3′, β-actin-R (antisense) 5′-AGA TGG GCA CAG TGT GGG-3′. RT-PCR products were separated by agarose gel electrophoresis and visualized using a UV illuminator.

### Enzyme-linked immunosorbent assay (ELISA) for OPG

The concentration of OPG in the culture medium was determined by use of a RayBio^®^ Human Osteoprotegerin ELISA kit (RayBiotech, Norcross, GA, USA). PC3-mock and PC3-OPG cells were seeded at 4×10^5^ cells/2 ml of medium/well onto 6-well plates and incubated for 72 h at 37°C in the presence of 1 mg of G418. After incubation, culture media were collected, centrifuged to remove cell debris and pre-diluted 100-fold before ELISA. These experiments were performed in duplicate. All values are means ± SD.

### Tartrate-resistant acid phosphatase (TRAP) assay

Mouse monocytes (Primary Cell, Sapporo, Japan) were plated in 96-well plates at 4×10^4^ cells/well. After pre-incubation over-night, cells were treated with 100 *μ*l supernatants from cultured PC3-mock or PC3-OPG cells (72-h culture at 4×10^5^ cells/2 ml) for 10 days, in the presence of 10 ng/ml M-CSF and RANKL (R&D Systems, Inc., Minneapolis, MN, USA). After incubation, cells in plates were washed with PBS, fixed with 3.7% phosphate-buffered formaldehyde and permeabilized with 100% ethanol. These cells were then stained with TRAP-staining solution [(5 mg of Naphtol AS-MX phosphate and 30 mg of Fast red violet LB salt (Sigma, St. Louis, MO, USA) in 50 ml of TRAP buffer (50 mM sodium tartrate and 45 mM sodium acetate)] and stained osteoclasts were counted under a microscope. Experiments were performed in n=6 and repeated twice. All values are means ± SD.

### Bone metastasis model in nude mice

Intratibial injections of PC3-mock, PC3-OPG or equal mixtures of PC3-GFP with PC3-mock or PC3-OPG were performed as described previously (21). Briefly, PC3-mock and PC3-OPG cells (5×10^5^ cells/mouse) were trypsinized, washed once in PBS and resuspended in Ca^2+^ and Mg^2+^-free Hank’s balanced salt solution at 5×10^5^ cells/20 *μ*l. For injection of mixed prostate cancer cells, suspensions of PC3-GFP, PC3-mock and PC3-OPG cells were prepared at 2.5×10^5^ cells/10 *μ*l and equal amounts of the PC3-GFP suspension and suspensions of PC3-mock or PC3-OPG (totally 5×10^5^ cells/mouse) were mixed. Prior to the intratibial injections, the mice were anesthetized with a ketamine hydrochloride (12 mg/ml)/xylazine (8 mg/ml) mixture. After precutaneous intraosseal injection was performed by drilling a 26-gauge needle into the tibia proximal to the tuberositas tibia, a 20-*μ*l tumor cell suspension was further inserted. After 9 weeks of observation, the mice were anesthetized, placed in a prone position and their hind limbs were subjected to radiography after which the animals were sacrificed and tumor incidence, the weight of the leg (excised at the knee joint) and the presence of intraabdominal lymph node metastases (incidence) were recorded. Bone tumors formed after the injection of mixed prostate cancer cells were partially used for microarray analysis.

### Histological analyses

After measuring the weight, the legs were fixed in 10% phosphate-buffered formalin for 24 h at room temperature, washed with PBS for 30 min and decalcified with 10% EDTA (pH 7.4) for 14 days at 4°C. Tissues were then embedded in paraffin and sectioned at 4–6 *μ*m. Detection of osteoclasts was conducted by TRAP-staining. Sections were deparaffinized with xylene, dehydrated with ethanol, washed with PBS and stained with TRAP-staining solution for 1 h at 37°C. Subsequently, sections were washed with PBS and counterstained with hematoxylin. Osteoclast density was calculated based on the number of TRAP-positive cells per 1 mm of bone tissue contacting the tumor region. Immunocytochemical staining was performed using the ChemMate EnVision kit/horseradish peroxidase (DakoCytomation, Carpenteria, CA, USA). Mouse monoclonal antibody for Ki-67 (DakoCytomation, Glostrup, Denmark), rabbit monoclonal antibodies for GFP (Cell Signaling, Danvers, MA) and RBP4 (Abcam, Cambridge, MA) and rabbit polyclonal antibodies for PLAC8 (Abcam) were used as primary antibodies. Sections were heated in 0.01 M citrate buffer (pH 6.0) for 10 min in a pressure cooker for antigen retrieval. Primary antibodies were added to the slides at a dilution of 1:50 and incubated for 1 h at room temperature. After washing with PBS, each slide was treated with horseradish peroxidase-conjugated secondary antibody for 40 min. Visualization was completed using 3,3′-diaminobenzidine and the sections were counterstained with Mayer’s hematoxylin (Muto Pure Chemicals, Tokyo, Japan).

### Microarray analysis

Bone tumors formed after the injection of mixed prostate cancer cells were minced with a sharp-edged knife and cultured for 1 week in MEM supplemented with 10% fetal bovine serum. The cells were then harvested using 1 mM EDTA. PC3-GFP cells were isolated from the mixture that included PC3-mock or PC3-OPG using fluorescence-activated cell sorting (FACS) and the EPICS^®^ XL-MCL (Beckman Coulter, Fullerton, CA, USA). Total RNA from PC3-GFP cells was isolated using an RNeasy Mini kit (Qiagen, Valencia, CA, USA). The relative purity of the RNA was measured using an Agilent 2100 Bioanalyzer (Agilent Technologies, Santa Clara, CA, USA). RNA expression was analyzed using a GeneChip^®^ Human Gene 1.0 ST Array (Affymetrix, Santa Clara, CA, USA) that contains 28,869 oligonucleotide probes for known and unknown genes. First strand cDNA was synthesized from 300 ng of total RNA using GeneChip Whole Transcript (WT) cDNA Synthesis and Amplification kit (Affymetrix) according to the manufacturer’s protocol. cRNA (10 *μ*g) were added into the second-cycle cDNA reaction and this cDNA was fragmented and end-labeled with the GeneChip WT Terminal Labeling kit (Affymetrix). Approximately 5.5 *μ*g of labeled DNA target was hybridized to the Affymetrix GeneChip Human Gene 1.0 ST Array at 45°C for 17 h on a GeneChip Hybridization 640 (Affymetrix) according to the manufacturer’s instructions. Hybridized arrays were washed and stained on a GeneChip Fluidics Station 450 and scanned using a GeneChip Scanner 3000 7G (Affymetrix), after which CEL files were generated for each array. This analysis was supported by the Support Center for Advanced Medical Sciences, the University of Tokushima Graduate School, Institute of Health Biosciences.

### RNAi interference

PC-3 cells were seeded on 60-mm dishes and preincubated overnight at 37°C. The following day, the cells were transfected with negative universal control siRNA (Invitrogen), RBP-4 siRNA (ID SASI_Hs01_00141628; Sigma) and PLAC8 siRNA (ID SASI_Hs01_00127245; Sigma) with Lipofectamine RNAiMAX (Invitrogen) according to the manufacturer’s protocol. After transfection, cells were reseeded on 96-well microplates (5×10^3^ cells/well) and preincubated overnight at 37°C. The culture medium was then replaced with 100 *μ*l of fresh complete medium and viable cells were counted by the 3-(4,5-dimethylthiazol-2-yl)-2,5-diphenyltetrazolium bromide (MTT) method after incubation for 24 h. Total RNAs from surplus cells were also isolated and RT-PCR analysis was conducted to confirm the effects of knockdown. The conditions for RT-PCR were described in ‘Semi-quantitative RT-PCR’. The sequences of specific primers for RBP-4 and PLAC8 were as follows: RBP-4-F (sense) 5′-GAGCGCGACTGCCGAGTGAG-3′, RBP-4-R (antisense) 5′-TCGAGGTTCAGGAGGCGGCA-3′, PLAC8-F (sense) 5′-TCTCCCAGGCCACAAGACATTTCC-3′ and PLAC8-R (antisense) 5′-CGGACCGGGACCGACTCCAG-3′.

### Statistical analyses

The two-tailed Student’s t-test was employed for analysis of OPG ELISA, MTT assay, *in vitro* TRAP assays, comparison of the average weights of hind legs, densities of osteoclasts and Ki-67 indices. Fisher’s exact probability test was used for comparison of the incidence of lymph node metastasis. In all cases, a probability level of less than 0.05 (P<0.05) was considered significant.

## Results

### Characteristics of PC3-OPG

**Figure 1:**
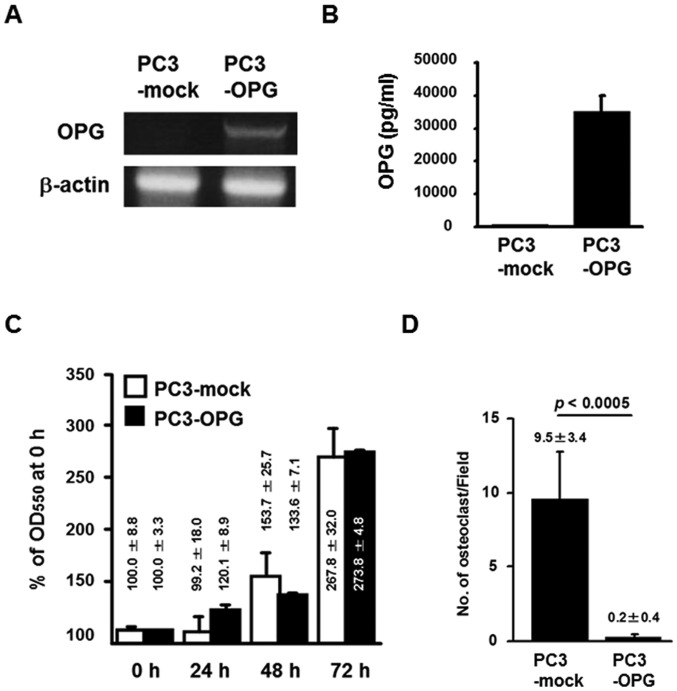
Characteristics of PC3-OPG. (A) Total RNA from PC3-mock and PC3-OPG cells was reverse-transcribed and the obtained cDNA was subjected to PCR for OPG and β-actin. PCR conditions were the same as those used for subcloning OPG cDNA, except for the number of amplification cycles (30 cycles). (B) The concentration of secreted OPG in the culture media was determined by OPG ELISA. (C) Cells were plated onto 96-well plates (5×10^3^ cells/well) and pre-incubated overnight. The cells were then further incubated for 24, 48 and 72 h and the growth rates were evaluated using the MTT method (n=6). (D) Mouse monocytes were incubated in PC3-mock- or PC3-OPG-cultured medium for 10 days, in the presence of RANKL and M-CSF. Differentiation into osteoclasts (TRAP-positive cells) was inhibited in the presence of PC3-OPG-cultured medium. Images are representative TRAP-positive cells stained red (left, monocyte incubated in PC3-mock-culture medium; right, monocyte incubated in PC3-OPG-culture medium). Values are means ± SD.

Using a PC-3 cell line, we established transfected cells that highly express OPG. The level of OPG mRNA in PC3-OPG cells, assessed by RT-PCR, was higher than that in the control vector-transfected cells, PC3-mock (Fig. 1A). This RT-PCR reaction was conducted under semi-quantitative conditions with 25 cycles of amplification. The level of OPG protein secreted into the culture media as measured by ELISA, was also higher in PC3-OPG cells than in PC3-mock cells. Indeed, culture media from PC3-mock contained 154±31 pg/ml OPG, whereas the level of OPG in PC3-OPG cells was 225-fold greater than that in PC3-mock cells (34,772±5,720 pg/ml, Fig. 1B). The growth rate of these cells was examined by the MTT assay, by calculating the percentage (%) of the OD_550_ in 0 h-cultured cells. There were no significant differences at any of the time-points measured (24-, 48- and 72-h-culture) (Fig. 1C).

### Inhibitory effect of culture supernatant from PC3-OPG cells on the differentiation of mouse monocytes to osteoclasts

Supernatants from 3-day cultures of transfectants were tested to determine whether they could influence differentiation of mouse monocytes into osteoclasts. Detection of osteoclasts was performed by the TRAP-staining method. The number of osteoclasts per field was 9.5±3.4 in the group treated with supernatants from PC3-mock cells. In contrast, a significant inhibition of osteoclastogenesis was found in the group treated with supernatants from PC3-OPG cells and the number of osteoclasts per field was 0.2±0.4 (P<0.0005, Fig. 1D).

### Effect of OPG on the development of prostate cancer cells in bone

**Figure 2:**
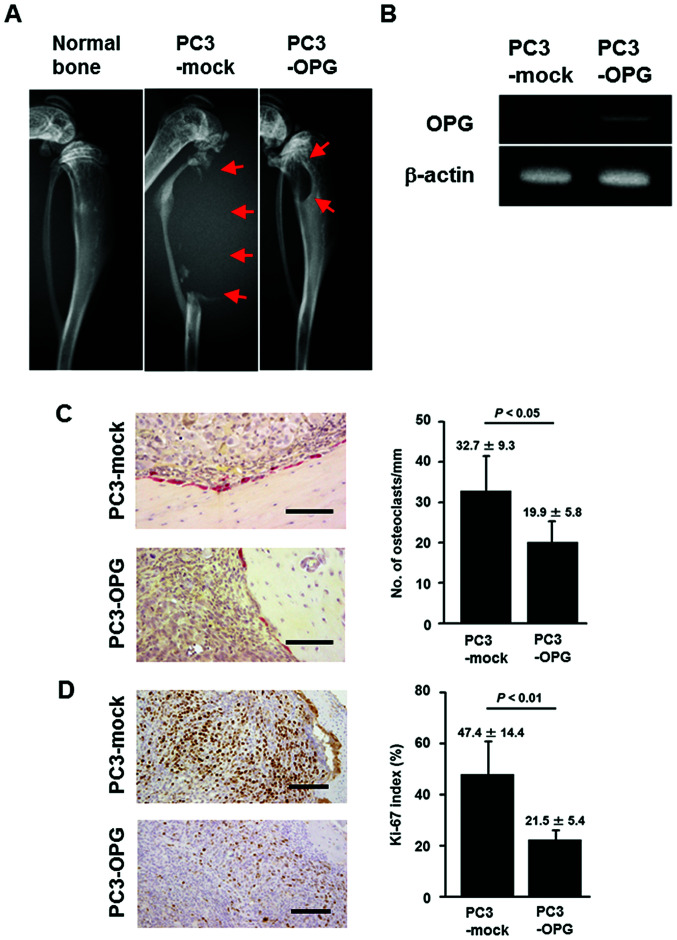
Effect of OPG overexpression on the growth of prostate cancer cells in the bones of nude mice. (A) Representative X-ray radiographs of normal, PC3-mock- or PC3-OPG-injected tibial bones of nude mice at the end of 9 weeks. Red arrow, bone lesion. (B) By using a part of the tumor tissue (9 weeks after injection of PC3-mock and PC3-OPG cells, respectively), semi-quantitative RT-PCR was conducted for OPG and β-actin. (C) Formalin-fixed paraffin-embedded sections of bone lesions were subjected to TRAP staining and the number of osteoclasts (TRAP-positive cells) per mm of tumor-contacting bone were counted. Values are means ± SD. Images are representative TRAP-positive cells stained red. Scale bar, 100 *μ*m. (D) Formalin-fixed paraffin-embedded sections of bone lesions were subjected to immunohistochemical staining using anti-Ki-67 antibody. Positive cells were counted and calculated to generate Ki-67 indices. Values are means ± SD. Images are representative Ki-67-stained cells. Scale bar, 200 *μ*m.

We injected PC3-mock or PC3-OPG cells into the bones of nude mice. After 5 weeks, osteolytic lesions were observed radiographically in 2 out 5 mice in the PC3-OPG-injected group. Conversely, all of the mice in the PC3-mock-injected group had lytic bone lesions (data not shown). Furthermore, after 9 weeks, tumor growth and severe osteolysis were observed in PC3-mock-injected mice, whereas the bone structure of PC3-OPG-injected mice was still preserved (Fig. 2A). At week 9, expression of OPG mRNA was only detected in bone tumors from PC3-OPG-injected mice (Fig. 2B). Although bone lesions were observed in all mice, the average weight of the hind legs and the incidence of intraabdominal lymph node metastases were significantly reduced in PC3-OPG-injected mice (P<0.05, Table I). TRAP staining of bone lesions showed that the density of osteoclasts on the bone surface facing the tumor was significantly reduced in PC3-OPG-injected mice (19.9±5.8 per mm bone) compared to PC3-mock-injected mice (32.7±9.3 per mm bone; P<0.05, Fig. 2C). The number of Ki-67-positive cells in the tumors was also reduced in PC3-OPG-injected mice (47.4±14.4% in PC3-mock-injected mice compared to 21.5±5.4% in PC3-OPG-injected mice; P<0.01, Fig. 2D).

### Analysis of gene expression in prostate cancer cells remaining in bone following osteolytic tumor growth inhibition by OPG

**Figure 3:**
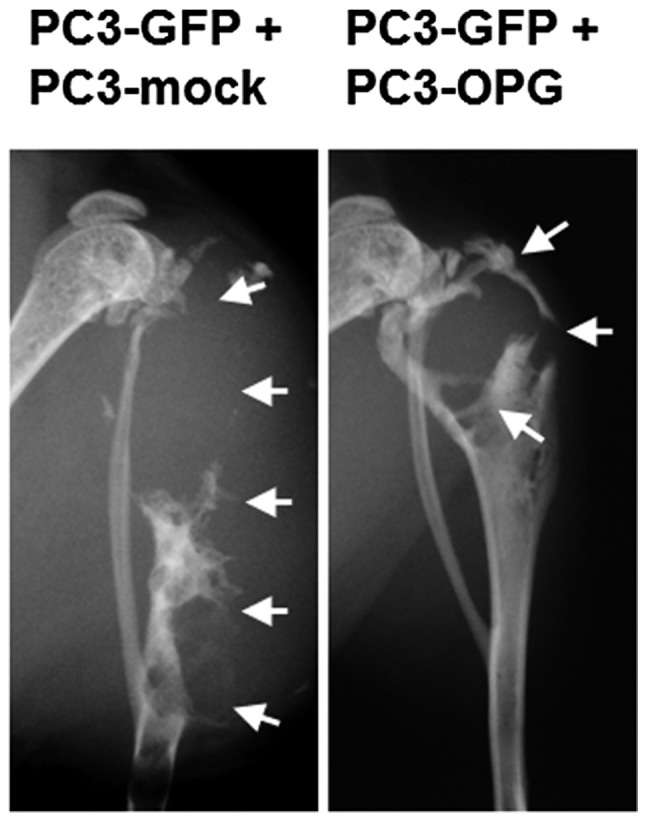
Effect of OPG overexpression on the growth of prostate cancer cells that were equally mixed with GFP-expressing prostate cancer cells in the bones of nude mice. Representative X-ray radiographs of bone lesions resulting from tibial bone injection of a mixture containing equal amounts of PC3-GFP and PC3-mock or PC3-OPG in nude mice at the end of 9 weeks. White arrow, bone lesion.

For the detection of genes that were up/downregulated in prostate cancer cells remaining in bone following osteolytic tumor growth inhibition by OPG, a mixture containing equal amounts of PC3-GFP and either PC3-OPG or PC3-mock, was injected into the bones of nude mice. At week 9, similar to the results seen with PC3-OPG injection, bone structure was preserved in mice injected with the PC3-GFP/PC3-OPG mixture compared to those injected with the PC3-GFP/PC3-mock mixture (Fig. 3). PC3-GFP cells isolated from tumors from each group were used for microarray analysis. The top 10 genes that were up/downregulated in PC3-GFP cells that maintained in a high-OPG bone microenvironment are shown in Table II. The gene showing the greatest upregulation was insulin-like growth factor binding protein 5 (fold change 5.02) and the most downregulated gene was transforming growth factor, β-induced (fold change 0.12). All genes in Table II displayed >3-fold differential expression compared to control.

### Increase of RBP4 and PLAC8 expression in prostate cancer cells remaining in bone following osteolytic tumor growth inhibition by OPG and the effect of RBP4 and PLAC8 knock-down on the growth of prostate cancer cells

**Figure 4:**
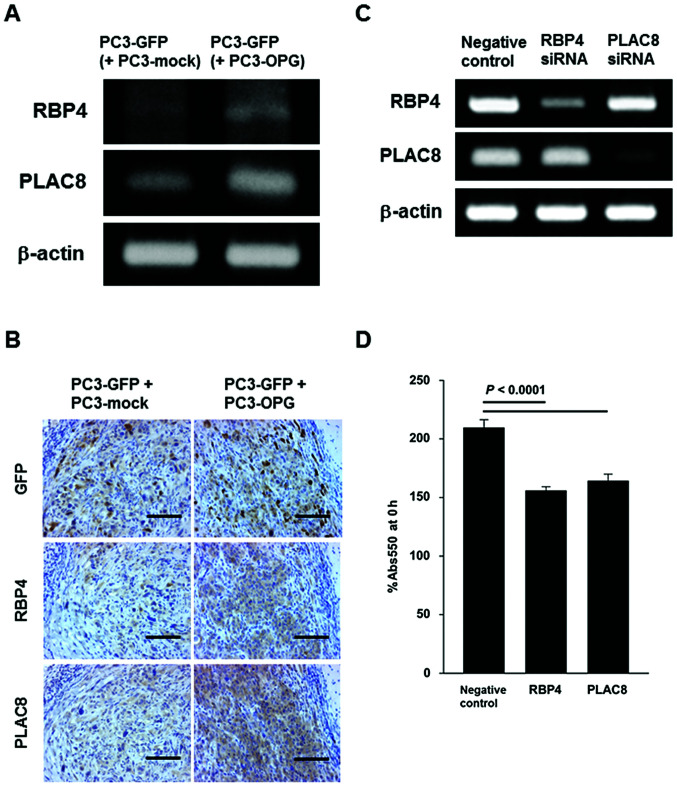
Increased expression of RBP4 and PLAC8 in prostate cancer cells remaining in bone of nude mice following osteolytic tumor growth inhibition by OPG and the effect of RBP4 and PLAC8 knockdown on the growth of prostate cancer cells. (A) Bone tumors were minced with a sharp-edged knife and cultured for 1 week. PC3-GFP cells were then separated from growing cells by a cell sorter. Total RNA from PC3-OPG cells was reverse-transcribed and the obtained cDNA was subjected to PCR for RBP4, PLAC8 and β-actin. (B) Formalin-fixed paraffin-embedded sections of bone lesions were subjected to immunohistochemical staining for GFP, RBP4 and PLAC8. Scale bar, 100 *μ*m. (C) PC-3 cells were transfected with negative control siRNA, RBP4 siRNA and PLAC8 siRNA. Specific knockdown of RBP4 and PLAC8 was confirmed. (D) Transfectants were plated onto 96-well plates (5×10^3^ cells/well) and pre-incubated overnight. Subsequently, cells were further incubated for 24 h and the growth rates were evaluated using the MTT method (n=6).

To examine the effect of knockdown of target genes by siRNA, we focused on the upregulated genes in Table II and confirmed mRNA expression of each gene by RT-PCR. Consequently, the expression patterns of RBP4 and PLAC8 were consistent with microarray results (Fig. 4A). Immunohistochemical staining of GFP, RBP4 and PLAC8 showed that bone tumors formed by injection of the mixture of PC3-GFP/PC3-mock or PC3-GFP/PC3-OPG, contained GFP-positive cells and the expression levels of both RBP4 and PLAC8 were increased in tumor cells from PC3-GFP/PC3-OPG-injected mice (Fig. 4B). Next, knockdown of RBP4 and PLAC8 by siRNA was performed in PC-3 cells. Each siRNA resulted in specific knockdown of the target gene without affecting expression of other genes (Fig. 4C). Knockdown of RBP4 and PLAC8 significantly inhibited the growth of PC-3 cells *in vitro* (P<0.0001, Fig. 4D).

## Discussion

Although to date there are no aggressive therapies for bone metastasis, a valid strategy that targets the underlying molecular mechanisms of bone metastasis, has recently been developed.

Bisphosphonates including zoledronic acid, are frequently administered to delay or prevent SREs (8,22). Moreover, denosumab, the first fully human monoclonal antibody against RANKL that mimics the endogenous effect of OPG, was shown to be effective in reducing skeletal morbidity rate in breast and prostate cancer (23–26) and found to be more effective in delaying the time to first SREs and reducing the risk of first and subsequent SREs compared to zoledronic acid (27–29). Thus, new therapies aimed at decreasing osteoclast activity including inhibition of the OPG/RANKL/RANK pathway, have demonstrated efficacy in the treatment of bone metastasis. There are, however, toxicities that have been reported to be associated with use of both denosumab and bisphosphonate (19,20), including osteonecrosis of the jaw and hypocalcemia, that may adversely affect therapies using these drugs. It is therefore necessary to identify additional therapeutic targets that can be combined with this type of therapy. In the present study, we demonstrated that OPG reduces osteolysis and cancer cell growth in a mouse model of bone metastasis and that expression of RBP4 and PLAC8, which were suggested to be associated with prostate cancer cell growth *in vitro*, was increased in maintained prostate cancer cells.

First, we examined whether expression of OPG in the androgen-independent human prostate cancer cell line PC-3, inhibits osteolysis and cancer cell growth in a mouse model of bone metastasis. Previous reports on the effects of OPG on bone metastasis of prostate cancers, were conducted using androgen-sensitive cells, although advanced prostate cancer eventually becomes androgen-independent (30). PC-3 differs from other androgen-sensitive cells not only in androgen receptor status, but also in status of p53 and in sensitivity to 1α,25-dihydroxyvitamin D3 and docetaxel (31–33). Furthermore, OPG has been reported to protect apoptosis of PC-3 cells induced by TRAIL (17) and promote angiogenesis (18). To generate an experimental system that enabled us to investigate effects of OPG, we established a PC-3-derived stable transfectant that highly expresses OPG, termed PC3-OPG. The relative expression levels of OPG mRNA and protein were clearly elevated in PC3-OPG cells compared with PC3-mock cells. In particular, a drastic increase (∼225-fold compared with PC3-mock cells) in the level of OPG secretion was observed. Since it is reported that the constitutive expression level of OPG in non-treated PC-3 cells is ∼750pg/ml (17), PC3-OPG can be regarded as a high-expressional subline derived from PC-3. By testing the growth rate of cells using the MTT method, we observed that the introduction of OPG cDNA was not cytotoxic. Similarly, a separate study showed no difference in the control of growth rates in another line of stable transfectants of OPG in C4-2 cells (9). The level of OPG secretion in that study was 8 ng/ml/10^6^ cells. In contrast, our established transfectant expresses OPG protein at ∼30 ng/ml/0.4×10^6^ cells. This suggests that high expression of this factor is not fatal to cell proliferation *in vitro*. To test the effect of PC3-OPG-cultured medium on differentiation of monocytes into mature osteoclasts, we conducted an *in vitro* TRAP assay. Treatment of cells with PC3-OPG-cultured medium inhibited the generation of TRAP-positive cells indicating that the OPG from PC3-hOPG cells is functionally effective as a decoy receptor for RANKL.

Administration of exogenous recombinant Fc-OPG protein was shown to inhibit tumor growth in bone (15), whereas implantation of breast cancer cells that overexpressed OPG aggravated tumor growth (34). Another study, however, reported that the prostate cancer-derived OPG-overexpressing cell line OPG-C4-2 grew more slowly in bone compared to vector control cells (14). Similarly, our results from the present study showed that the growth of PC3-OPG cells in bone was almost completely inhibited. At 9 weeks, tumors in the PC3-mock-injected group continued to develop and absolute osteolysis was seen in all mice. In contrast, no similar lesions were observed in PC3-OPG-injectd mice. In addition, the average weight of the hind legs and the incidence of intraabdominal lymph node metastasis were significantly decreased in PC3-OPG-injected mice. Moreover, histological examination showed that the density of osteoclasts on the bone surface facing the tumor, as well as the number of Ki-67-positive cells in the tumors were reduced in PC3-OPG-injected mice. These results suggest that OPG acts mainly as an inhibitor of the growth of PC-3 cells that is associated with the surrounding bone environment. Its observed effects on protection from apoptosis in PC-3 cells and the promotion of angiogenesis, are limited *in vivo*.

To find new therapeutic targets associated with inhibition of the OPG/RANKL/RANK pathway, we injected mixtures containing equal amounts of PC3-GFP and PC3-mock or PC3-OPG cells into the bones of nude mice and analyzed PC3-GFP cells, which are not affected by internal OPG over-expression, using cDNA microarray, Bone destruction was reduced even by injection of the PC3-GFP/PC3-OPG mixture, compared to PC3-GFP/PC3-mock mixture. Tumor cells were cultured once because of the difficulty in separating tumors into single cells for sorting. After being cultured for 1 week, PC3-GFP cells were isolated and cDNA microarray analyses were performed. As shown in Table II, all of the top 10 genes that were up/downregulated in PC3-GFP cells following osteolytic tumor growth inhibition by OPG, were differentially expressed >3-fold, compared to control. We focused on upregulated genes in order to examine the effect of knockdown of target genes by siRNA. When mRNA expression was confirmed in each gene by RT-PCR, however, only RBP4 and PLAC8 showed the same expression pattern seen in the results from the microarray. Since there is a possibility that one week culturing may affect gene expression of PC3-GFP, expression levels of GFP, RBP4 and PLAC8 in bone tumors were examined by immunohistochemical staining. GFP-positive cells were observed in the tumors from both PC3-GFP/PC3-mock and PC3-GFP/PC3-OPG. On the other hand, the expression of RBP4 and PLAC8 was increased in tumor cells from mice injected with PC3-GFP/PC3-OPG in accordance with the results from RT-PCR and cDNA microarray analysis. These findings suggest that osteolytic tumor growth inhibition by OPG is associated with upregulation of RBP4 and PLAC8 in prostate cancer cells.

RBP4 belongs to the lipocalin family that transport small hydrophobic molecules (35) and transport retinol from the liver to peripheral tissues (36). The liver has the highest expression level of RBP4 (37), which has been shown to be a promising adipokine linking adiposity and insulin resistance in animal models and humans (38–41). Indeed, proteome analysis shows that serum RBP4 is a potential new biomarker of lung and pancreatic cancer (42,43). A direct relationship between RBP4 and cancer, however, has not been elucidated.

PLAC8, also known as onzin, is a small, highly conserved, cysteine-rich protein that is a negatively-regulated target of the c-Myc-gene. It affects cell growth and apoptosis and is highly expressed in myeloid cells and pancreatic cancer cells. Induced expression of onzin in fibroblasts promotes growth, loss of cell cycle control, resistance to apoptosis and tumorigenesis. In contrast, inhibition of endogenous expression of onzin, reduces cell growth and increases sensitivity to apoptotic stimuli (44–46).

Knockdown of RBP4 and PLAC8 in PC-3 cells by siRNA in the present study, resulted in significant inhibition of growth of PC-3 cells *in vitro*. This result of PLAC8 knockdown is consistent with previous reports (46). In contrast, molecular mechanisms for regulation of cancer cell growth by RBP4 must be clarified in future studies. Since the expression of RBP4 and PLAC8 were increased in tumor cells in PC3-GFP/PC3-OPG-injected mice compared to controls, RBP4 and PLAC8 may be acting as survival factors in prostate cancer cells when osteolytic tumor growth is inhibited by OPG.

In conclusion, OPG reduced osteolysis and cancer cell growth in a mouse model of bone metastasis. In addition, the expression of RBP4 and PLAC8, which were suggested to be associated with prostate cancer cell growth *in vitro*, was increased in remaining prostate cancer cells in bone. RBP4 and PLAC8 may become new therapeutic targets for prostate cancer bone metastasis, to be used in combination with therapies that inhibit the OPG/RANKL/RANK pathway.

## Figures and Tables

**Table I:** Tumor incidence, weight of hind legs and lymph node metastasis in mice injected with PC3-mock or PC3-OPG.^a^

	Incidence of bone tumors	Average weight of hind legs (g)	Incidence of LN metastasis^b^
PC3-mock	100% (5/5)	1.06±0.29	100% (5/5)
PC3-OPG	100% (5/5)	0.71±0.07^c^	20% (1/5)^d^

a This analyses were done at the end of 9-week observation.

b LN, lymph node. Significantly different from PC3-mock at P<0.05 by Student’s t-test.

c Significantly different from PC3-mock at P<0.05 by Fisher’s exact probability test.

d Significantly different from PC3-mock at P<0.05 by Fisher’s exact probability test.

**Table II:** Top 10 up/downregulated genes in PC3-GFP cells that maintained in a high-OPG bone microenvironment, identified by cDNA microarray analysis.

GenBank accession no.	Gene name	Fold change^a^
Upregulated genes		
NM_000599	Insulin-like growth factor binding protein 5 (IGFBP5)	5.02
NM_020775	KIAA1324 (KIAA1324)	4.67
NM_000096	Ceruloplasmin (ferroxidase) (CP)	3.61
NM_024727	Leucine rich repeat containing 31 (LRRC31)	3.59
NM_032192	Protein phosphatase 1, regulatory (inhibitor) subunit 1B (PPP1R1B), transcript variant 1	3.58
NM_001080505	Shisa homolog 3 (*Xenopus laevis*) (SHISA3)	3.41
NM_006744	Retinol binding protein 4, plasma (RBP4)	3.25
NM_001145029	Ankyrin repeat domain 30B (ANKRD30B)	3.21
NM_003657	Breast carcinoma amplified sequence 1 (BCAS1)	3.13
NM_016619	Placenta-specific 8 (PLAC8), transcript variant 2	3.02
Downregulated genes		
NM_000358	Transforming growth factor, β-induced, 68 kDa (TGFBI)	0.12
NM_001143818	Serpin peptidase inhibitor, clade B (ovalbumin), member 2 (SERPINB2), transcript variant 1	0.13
NM_014439	Interleukin 1 family, member 7 (ζ) (IL1F7), transcript variant 1	0.22
NM_000963	Prostaglandin-endoperoxide synthase 2 (prostaglandin G/H synthase and cyclooxygenase) (PTGS2)	0.23
NM_002993	Chemokine (C-X-C motif) ligand 6 (granulocyte chemotactic protein 2) (CXCL6)	0.26
NM_005554	Keratin 6A (KRT6A)	0.27
NM_004385	Versican (VCAN), transcript variant 1	0.27
NM_001013398	Insulin-like growth factor binding protein 3 (IGFBP3), transcript variant 1	0.29
NM_018099	Fatty acyl CoA reductase 2 (FAR2)	0.29
NM_002994	Chemokine (C-X-C motif) ligand 5 (CXCL5)	0.30

a The microarray results of PC-3-GFP cells co-injected with PC-3-OPG cells were divided by those of cells co-injected with PC-3-mock.
